# Longitudinal Surveillance of Fetal Heart Failure Using Speckle Tracking Analysis

**DOI:** 10.3390/jcm11237102

**Published:** 2022-11-30

**Authors:** Julia Murlewska, Oskar Sylwestrzak, Maria Respondek-Liberska, Mark Sklansky, Greggory Devore

**Affiliations:** 1 Cardiology Department, Polish Mother’s Memorial Hospital, Research Institute, 93-338 Lodz, Poland; 2 Department for Fetal Malformations Diagnoses & Prevention, Medical University of Lodz, 93-338 Lodz, Poland; 3 Division of Pediatric Cardiology, Department of Pediatrics, UCLA Mattel Children's Hospital, David Geffen School of Medicine at UCLA, Los Angeles, CA 90095, USA; 4 Division of Maternal-Fetal Medicine, Department of Obstetrics and Gynecology, David Geffen School of Medicine at UCLA, Los Angeles, CA 90095, USA; 5 Fetal Diagnostic Centers, Pasadena, CA 91105, USA

**Keywords:** fetal echocardiography, fetal strain, fetal speckle tracking, fetal heart failure

## Abstract

Long-term monitoring of a fetus with heart failure is an undeniable challenge for prenatal cardiology. Echocardiography is constrained by many fetal and maternal factors, and it is difficult to maintain the reproducibility of the measured and analyzed parameters. In our study, we presented the possibilities of using modern speckle tracking technology in combination with standard echocardiography parameters that may be insufficient or less sensitive in the context of monitoring life-threatening fetal conditions. Our analysis shows the superiority of the parameters used to assess fetal cardiac architecture, such as the GSI Global sphericity Index, and fetal cardiac function, such as the FAC fractional area change and the EF ejection fraction, which temporal change may indicate a worsening condition of the fetus with heart failure. The significant increase in the parameters of fetal heart size in speckle tracking allows for an improved echocardiographic diagnosis and monitoring of the fetus with heart failure and the prognostic conclusions about the clinical condition after birth. Significant decreases in FAC for the left and right ventricles and EF for the left ventricle may indicate an unfavourable prognosis for the monitored fetus due to heart failure.

## 1. Introduction

**Figure 1:**
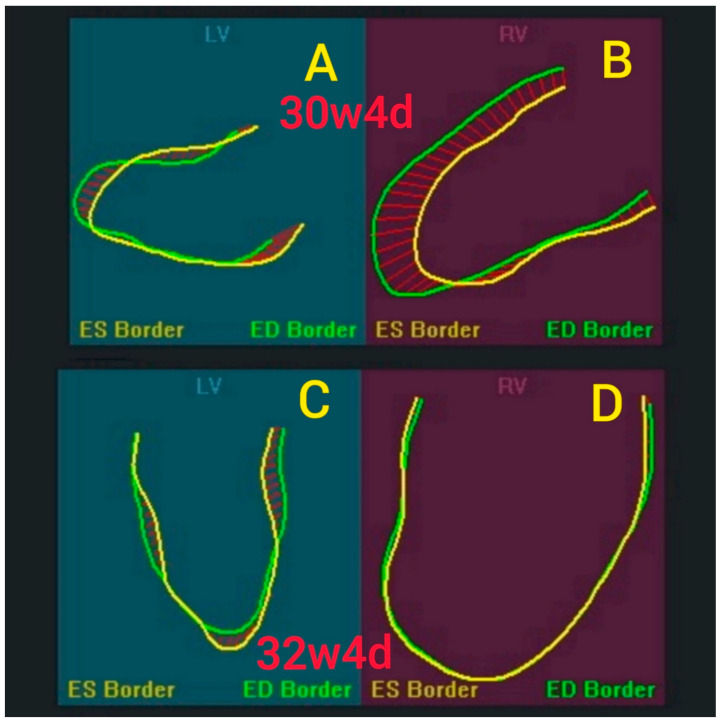
(**A**,**B**) Fetal strain at 30 weeks 4 days. (**C**,**D**) Fetal strain at 32 weeks 4 days. End-Systolic/End-Diastolic Borders of LV-left ventricle and RV-right ventricle displacement.

For longitudinal echocardiographic monitoring during fetal life, there is no consensus on the best approach to evaluate fetal cardiovascular function. Compared to postnatal echocardiographic evaluation, fetal evaluations may be complicated by various fetal positions and orientations, making serial assessment with a single measurement more challenging. Therefore, we compared conventional echocardiographic measurements with speckle-tracking derived assessments [1] (Figure 1) to determine which approach, in a single fetus with progressive heart failure, may be most useful to predict fetal well-being.

## 2. Materials and Methods

During the first pregnancy of a 26-year-old woman, the first trimester evaluation demonstrated active herpes labialis and shingles, which were treated with Aciclovir. New onset of ascites, hydrops testis, and ductus venosus reversal flow was seen at 30 weeks of gestation. With the subsequent development of fetal cardiomegaly, cardiac hypertrophy, pericardial effusion, mitral, tricuspid, and pulmonary valve regurgitation, and fetal heart failure, transplacental digoxin treatment was initiated, along with dexamethasone and cefuroxime. The standard echo-sonographic findings are presented in Table 1. Speckle tracking analysis (Table 2, video S1) has been demonstrated to provide both qualitative and quantitative assessments of fetal size, shape, and function, as recently described by DeVore [1,2,3,4]. Speckle tracking forms a characteristic pattern of spots and kernels on the irregular surfaces of fetal myocardium to measure longitudinal, radial, and circumferential regional strain [5]. Pregnancy was maintained for over two more weeks, but was then complicated by polyhydramnios, maternal hypertension, and premature rapture of membrane. Due to abnormal fetal tracings, an emergency CS was performed at 32 weeks of gestation. The newborn baby boy had a birth weight of 2500 g, Apgars 5/6/6, and was admitted to the ICU. Following aggressive postnatal medical management, as well as PDA-patent ductus arteriosus ligation at 1 month of age, he was discharged home after 79 days with a diagnosis of congestive herpetic cardiomyopathy.

## 3. Results

Our analysis and interpretation of the standard and advanced echocardiographic parameters using speckle tracking technology allowed us to evaluate the efficacy of prenatal treatment with digoxin. Digoxin was administered from week 30 to week 32. Standard size of the HA/CA (heart area/chest area) was constant, and a detailed speckle tracking analysis revealed a significant prolongation of the TL transverse length dimension and a shortening of the BAL-basal–apical length dimension, thereby reducing the GSI-global Sphericity Index GSI = BAL/TL) [2]. We also observed an increase in the following parameters: the area and circumference of the heart and the area for the left and right ventricles. Standard cardiovascular parameters, such as the Tei index or MPI, for the left and right ventricles increased without significant difference over a two-week period (values between 0.7 and 0.9), and the CVPS cardiovascular profile score remained unchanged at level 5. Fetal global cardiac contractility was also analyzed using the parameters FAC-fractional area change (%) and EF-ejection fraction (%) [3,4], which significantly decreased over time (Table 1 and Table 2 show the analyzed echocardiographic parameters). The tables contain selected parameters that allow the analysis of fetal heart failure.

## 4. Discussion

Fetal circulatory failure may have various causes and, therefore, it is very difficult to assess the effectiveness of any treatment [6]. So far, cardiovascular profile score has been considered a very useful diagnostic tool to quantify the severity of heart failure, which is a composite score based on five different echocardiographic parameters [7]. Our long-term analysis presents a modern approach of monitoring a fetus with circulatory failure. We tried to show the superiority of speckle tracking analysis, which is a more detailed method; although it is more involved and time-consuming, it can have a very important place precisely for the studies of fetuses with the most severe cardiovascular and non-cardiovascular conditions that lead to circulatory failure and fetal death. Speckle tracking may indicate some changes in fetal cardiac architectonics not seen by standard echocardiography, and may also have superiority over standard fetal cardiac function exponents, which may be less sensitive than FAC and EF; we have highlighted these findings which seem very promising in the context of monitoring a fetus with circulatory failure. The pattern of speckles within a defined region, known as a kernel, can be conceptualized as an ‘acoustic fingerprint’. The spatial movement of multiple kernels can be used to measure longitudinal, radial, and circumferential regional strain. Changes in myocardial strain precede the changes in ejection fraction in patients with cardiomyopathy. STE is less dependent on the angle of insonation than conventional echocardiography, allowing more flexibility in image acquisition than Doppler-based techniques. STE is less susceptible to fetal and maternal movements. The accuracy of STE has been confirmed in postnatal life in animal models [5].

FAC should generally take values ≥35% [3], while EF should be >50% [4]. In our case, these values corresponded to <−2.93 Z-score and significantly decreased over a two-weeks below the mean, the CVPS was constant, and its analysis made it difficult to predict an impending premature birth. M-mode analysis of the myocardial shortening fraction can also be made more accurate using speckle tracking and 24-segment examination [8] Finally, a HA/CA analysis associated with fetal cardiomegaly may not be adequate for serious cases of fetuses with heart failure, in which more demanding and more sensitive parameters to the deterioration of circulatory performance, such as GSI, LV Area, or RV Area, could be used.

## 5. Conclusions

The speckle tracking analysis performed in our case allows a detailed monitoring of a fetus with heart failure.

## Figures and Tables

**Table 1:** Standard selected echocardiographic parameters analyzed in the present case indicating the progression of fetal heart failure.

GA	30 Weeks 4 Days	32 Weeks 4 Days
Cardiomegaly/HA/CA	0.4	0.4
Tei RV	0.9	0.9
Tei LV	0.7	0.7
RV SF	18%	16%
LV SF	24%	**17%**
CVPS	5	5

GA: gestational age; HA/CA: heart area/chest area; RV/Tei LV: MPI-myocardial performance index for right and left ventricle; RV/LV SF: shortening fraction for right and left ventricle; CVPS: cardiovascular profile score.

**Table 2:** Advanced echocardiographic parameters of the fetal heart using speckle tracking technology.

	30 Weeks 4 Days		32 Weeks 4 Days		
	Measurement	Z-Score	Measurement	Z-Score	
Global Sphericity Index (BAL/TL)	1.46	2.38	1.21	−0.22	↓
BAL-Basal–Apical Length (mm)	63.10	8.12	57.00	4.76	↓
TL-Transverse Length (mm)	43.20	5.02	47.00	5.19	↑
Area	1929.00	8.51	2177.00	6.23	↑
Circumference	164.00	7.60	171.00	5.39	↑
Left Ventricular Area (mm^2^)	3.22	2.89	3.60	2.91	↑
Right Ventricular Area (mm^2^)	4.59	8.28	4.43	6.06	
Right Ventricular Fractional Area Change (%)	5.68	−6.65	−16.22	−10.78	↓
Left Ventricular Fractional Area Change (%)	28.89	−2.97	12.97	−6.29	↓
Left Ventricular Ejection Fraction (%)	41.61	−2.93	24.61	−5.69	↓

## Data Availability

Not applicable.
